# Cloning strategies for heterologous expression of the bacteriocin enterocin A by *Lactobacillussakei* Lb790, *Lb. plantarum* NC8 and *Lb. casei* CECT475

**DOI:** 10.1186/s12934-015-0346-x

**Published:** 2015-10-15

**Authors:** Juan J. Jiménez, Dzung B. Diep, Juan Borrero, Loreto Gútiez, Sara Arbulu, Ingolf F. Nes, Carmen Herranz, Luis M. Cintas, Pablo E. Hernández

**Affiliations:** Departamento de Nutrición, Bromatología y Tecnología de los Alimentos, Facultad de Veterinaria, Universidad Complutense de Madrid (UCM), Avenida Puerta de Hierro, s/n, 28040 Madrid, Spain; Department of Chemistry, Biotechnology and Food Science, Norwegian University of Life Sciences (NMBU), P.O. Box 5003, 1432 Ås, Norway

**Keywords:** Bacteriocins, Enterocin A, Lactic acid bacteria (LAB), Expression systems, *Lactobacillus* spp., Heterologous bacteriocin production

## Abstract

**Background:**

Bacteriocins produced by lactic acid bacteria (LAB) attract considerable interest as natural and nontoxic food preservatives and as therapeutics whereas the bacteriocin-producing LAB are considered potential probiotics for food, human and veterinary applications, and in the animal production field. Within LAB the lactobacilli are increasingly used as starter cultures for food preservation and as probiotics. The lactobacilli are also natural inhabitants of the gastrointestinal (GI) tract and attractive vectors for delivery of therapeutic peptides and proteins, and for production of bioactive peptides. Research efforts for production of bacteriocins in heterologous hosts should be performed if the use of bacteriocins and the LAB bacteriocin-producers is ever to meet the high expectations deposited in these antimicrobial peptides. The recombinant production and functional expression of bacteriocins by lactobacilli would have an additive effect on their probiotic functionality.

**Results:**

The heterologous production of the bacteriocin enterocin A (EntA) was evaluated in different *Lactobacillus* spp. after fusion of the versatile Sec-dependent signal peptide (SP_*usp45*_) to mature EntA plus the EntA immunity gene (*entA* + *entiA*) (fragment UAI), and their cloning into plasmid vectors that permitted their inducible (pSIP409 and pSIP411) or constitutive (pMG36c) production. The amount, antimicrobial activity (AA) and specific antimicrobial activity (SAA) of the EntA produced by *Lactobacillus sakei* Lb790, *Lb. plantarum* NC8 and *Lb. casei* CECT475 transformed with the recombinant plasmids pSIP409UAI, pSIP411UAI and pMGUAI varied depending of the expression vector and the host strain. The *Lb. casei* CECT475 recombinant strains produced the largest amounts of EntA, with the highest AA and SAA. Supernatants from *Lb. casei* CECT (pSIP411UAI) showed a 4.9-fold higher production of EntA with a 22.8-fold higher AA and 4.7-fold higher SAA than those from *Enterococcus faecium* T136, the natural producer of EntA. Moreover, supernatants from *Lb. casei* CECT475 (pSIP411UAI) showed a 15.7- to 59.2-fold higher AA against *Listeria* spp. than those from *E. faecium* T136.

**Conclusion:**

*Lb. casei* CECT457 (pSIP411UAI) may be considered a promising recombinant host and cell factory for the production and functional expression of the antilisterial bacteriocin EntA.

## Background

Within lactic acid bacteria (LAB) the lactobacilli are increasingly used as starter cultures for food preservation and as probiotics [1]. The lactobacilli are also natural inhabitants of the gastrointestinal (GI) tract and attractive vectors for delivery of therapeutic peptides and proteins and production of bioactive peptides [2, 3]. Furthermore, most probiotics enhance intestinal barrier function, display immunomodulatory activity and exert protective effects against pathogens due to the production of antimicrobial compounds [4–6]. Since the in situ production of the antilisterial bacteriocin Abp118 is the major reason of the well-documented probiotic effect of *Lb. salivarius* UCC118 against *Listeria monocytogenes* EFDe infections in mice [7, 8], the production of bacteriocins by lactobacilli surely would have an additive effect on their probiotic functionality.

Bacteriocins are ribosomally synthesized antimicrobial peptides secreted by bacteria, and those produced by LAB attract considerable interest as natural and nontoxic food preservatives, for human and veterinary applications, and in the animal production field [9, 10]. Most bacteriocins, including those produced by enterococci and named enterocins are synthesized as biologically inactive precursors or prepeptides containing an N-terminal extension of the so-called double-glycine type (leader sequence) that is cleaved concomitantly with export across the cytoplasmic membrane by dedicated ATP-binding cassette transporters (ABC transporters) and their accessory proteins [11]. However, many secreted prokaryotic proteins and a few bacteriocins contain N-terminal extensions of the Sec-dependent type (signal peptide) that are proteolytically cleaved concomitantly with peptide externalization by the general secretory pathway (GSP) or Sec-dependent pathway [12]. And the signal peptide (SP) of secretory proteins and bacteriocins may drive fused mature bacteriocins to SPs for their secretion by recombinant LAB [9, 10, 13]. The mature bacteriocins are often cationic, amphiphilic molecules of 20–60 amino acid residues that are classified into two main classes: the lantibiotics or class I that consist of modified bacteriocins and the class II or nonmodified bacteriocins which are further subdivided in class IIa, class IIb, class IIc, and class IId. Among these subgroups, the class IIa bacteriocins (also referred to as pediocin-like bacteriocins) have attracted much attention due to their strong antilisterial activity [14]. Additional subgroups have been suggested for leaderless peptides, circular bacteriocins, linear peptides derived from large proteins, and the glycosylated bacteriocins [15].

Accordingly, bacteriocins with high antimicrobial activity against bacterial pathogens could be overproduced and would contribute to the probiotic effect of recombinant *Lactobacillus* spp. strains [8, 16]. Enterocin A (EntA) is a class IIa bacteriocin whose synthesis is directed by the *entAIFKRTD* operon and from which *entA* encodes the enterocin A prepetide synthesized as an 18 amino acid leader sequence of the double-glycine type and the 47 amino acid mature bacteriocin [17, 18]. Moreover, its potent antilisterial activity has driven interest for its overproduction by LAB mostly of the genera *Lactococcus*, *Enterococcus* and *Pediococcus* [13, 19] and also by yeasts from the genera *Pichia*, *Kluyveromyces*, *Hansenula* and *Arxula*, throughout fusions of mature EntA to signal peptides (SPs) that act as secretion signals [20, 21]. Accordingly, of biotechnological interest would be the design and construction of recombinant *Lactobacillus* spp. for the controlled or constitutive heterologous production of bacteriocins with high antimicrobial activity against *Listeria* spp.

In this work, *Lb. sakei* Lb790 a non-bacteriocin producing strain from meat origin [22], *Lb. plantarum* NC8 from grass silage encoding the two-peptide plantaricins PlnEF, PlnJK and PLNC8αβ of narrow inhibitory spectra [23, 24] and *Lb. casei* CECT475, a reported non-bacteriocin producer from dairy origin, were transformed with derivatives of the inducible protein expression vectors pSIP409 and pSIP411 and the constitutive pMG36c expression vector, for evaluation of the production of EntA and its functional expression as determined by evaluation of their antimicrobial activity against *Listeria* spp.

## Results

### Heterologous production and functional expression of EntA by different *Lactobacillus* spp. strains

Since the leader sequence of EntA (LS_*entA*_) is of a double-glycine type which restricts expression of the bacteriocin to limited LAB strains containing homologous dedicated ABC-transporters, we therefore employed the more versatile signal peptide SP_*usp45*_ for the Sec-dependent externalization of mature EntA, as well as the use of protein expression vectors that permitted the inducible (pSIP409, pSIP411) or constitutive (pMG36c) production of the synthesized bacteriocin by different *Lactobacillus* spp. host strains. Thus, cloning of the lactococcal SP_*usp45*_ fused to mature *entA* (EntA) and *entiA* (EntI) (fragment UAI) into plasmids pSIP409, pSIP411 and pMG36c resulted in the plasmid derived vectors pSIP409UAI, pSIP411UAI and pMGUAI, respectively. Transformation of *Lb. sakei* Lb790, *Lb. plantarum* NC8 and *Lb. casei* CECT475 with plasmids pSIP409UAI, pSIP411UAI and pMGUAI yielded recombinant *Lactobacillus* spp.-derived strains which were further checked by bacteriocinogenicity tests, PCR and sequencing of the inserts. Halos of inhibition of variable sizes were observed by all transformed *Lactobacillus* spp. (results not shown), confirming that recombinant plasmids were responsible of their antimicrobial activity.

The production and functional expression of the EntA in supernatants of the recombinant *Lactobacillus* spp. strains was quantified using specific anti-EntA antibodies in a NCI-ELISA, and by a microtitre plate assay (MPA). None of the native *Lactobacillus* spp. strains showed production of EntA (Table 1). The production of EntA by *Lb. sakei* Lb790 (pSIP411UAI) and *Lb. casei* CECT475 (pSIP411UAI) was 2.7- and 4.9-fold higher, respectively, whereas production of EntA by *Lb. plantarum* NC8 (pSIP411UAI) was 4.7-times lower than production of EntA by the natural producer *E. faecium* T136. The production of EntA by *Lb. sakei* Lb790, *Lb. plantarum* NC8 and *Lb. casei* CECT475 transformed with either pSIP409UAI or pMGUAI, was 1.1- to 6.3-times lower than production of EntA by *E. faecium* T136 (Table 1).

**Table 1:** Bacteriocin production and antimicrobial activity of supernatants from recombinant strains

Strain	Bacteriocin production (µg/mg cell dry weight)^a^	Antimicrobial activity (BU/mg cell dry weight)^b^	Specific antimicrobial activity (BU/µg EntA)^c^
*Lactobacillus sakei*			
Lb790	NP	NA	NE
Lb790 (pSIP409UAI)	1.3	324	249
Lb790 (pSIP411UAI)	5.2	1578	303
Lb790 (pMGUAI)	0.7	48	68
*Lactobacillus plantarum*			
NC8	NP	NA	NE
NC8 (pSIP409UAI)	0.4	42	105
NC8 (pSIP411UAI)	0.4	36	90
NC8 (pMGUAI)	0.3	19	63
*Lactobacillus casei*			
CECT475	NP	102	NE
CECT475 (pSIP409UAI)	1.7	958	1629
CECT475 (pSIP411UAI)	9.3	16,466	1771
CECT475 (pMGUAI)	1.1	869	790
*Enterococcus faecium*			
T136^d^	1.9	721	379

Most of the data are mean from two independent determinations in triplicate

*NP* no production, *NA* no activity, *NE* not evaluable

^a^Production of EntA was calculated by using a NCI-ELISA with polyclonal antibodies specific for EntA

^b^Antimicrobial activity was calculated against *E. faecium* P13 (EntA^s^). BU, bacteriocin units

^c^Specific antimicrobial activity refers to the antimicrobial activity against *E. faecium* P13 divided by the EntA produced

^d^Culture of *E. faecium* T136 used as control for production and antimicrobial activity of EntA

When supernatants of the recombinant *Lb. sakei* Lb790, *Lb. plantarum* NC8 and *Lb. casei* CECT475 strains were evaluated for their antimicrobial activity against *E. faecium* P13 (EntA^S^), the antimicrobial activity (AA) of *Lb. sakei* Lb790 (pSIP411UAI) was 2.2-fold higher while its specific antimicrobial activity (SAA) was 1.2-times lower than the EntA produced by *E. faecium* T136 (Table 1). *Lb. sakei* Lb790 (pSIP409UAI) showed 2.2-times lower AA and 1.5-times lower SAA and *Lb. sakei* Lb790 (pMGUAI) showed 15-times lower AA and 5.5-times lower SAA, when compared to the control EntA producer. All *Lb. plantarum* NC8 recombinants showed a 17.1- to 38-times lower AA and 3.6- to 6.0-times lower SAA, when compared to the control EntA producer. However, transformation of *Lb. casei* CECT475 with plasmids pSIP409UAI, pSIP411UAI and pMGUAI generated supernatants with 1.3-, 22.8- and 1.2-fold higher AAA and 4.3-, 4.7- and 2.1-fold higher SAA, respectively, than those from *E. faecium* T136 (Table 1).

Furthermore, the evaluation of the antimicrobial activity of supernatants from the recombinant *Lb. sakei* Lb790, *Lb. plantarum* NC8 and *Lb. casei* CECT475 against five *Listeria* spp. and six *L. monocytogenes* strains, showed that supernatants from *Lb. sakei* Lb790 (pSIP411UAI) displayed 3.8-times lower to 2.7-fold higher AA whereas those from *Lb. sakei* Lb790 (pSIP409UAI) and *Lb. sakei* Lb790 (pMGUAI) showed 1.8- to 9.1-times lower and a 6.0- to 45-times lower AA, respectively, than those from *E. faecium* T136. Supernatants from all recombinant *Lb. plantarum* NC8 strains showed a 4.9- to 120-times much lower AA than the control EntA producer (Table 2). However, despite the measurable and non-previously reported antimicrobial activity of *Lb. casei* CECT475, the supernatants from *Lb. casei* CECT475 (pSIP409UAI) showed 1.6- to 13.9-fold higher AA, those from *Lb. casei* CECT475 (pSIP411UAI) showed 15.7- to 59.2-fold higher AA and those from *Lb. casei* CECT475 (pMGUAI) showed 0.54- to 4.9-fold higher AA than those from *E. faecium* T136 (Table 2).

**Table 2:** Antimicrobial activity of supernatants from recombinant *Lactobacillus* spp. strains against *Listeria* spp.^a^

Strain	*L. ivanovii*	*L. grayi*	*L. welshimeri*	*L. seeligeri*	*L. innocua*	*L. monocytogenes*					
	CECT913	CECT931	CECT919	CECT917	CECT910	CECT911	CECT935	CECT936	CECT939	CECT4031	CECT4032
*Lactobacillus sakei*											
Lb790	NA	NA	NA	NA	NA	NA	NA	NA	NA	NA	NA
Lb790 (pSIP409UAI)	1450	2575	1797	2012	1019	1387	1254	3317	508	545	1427
Lb790 (pSIP411UAI)	5317	12,350	15,045	7866	6485	10,852	10,533	15,454	1661	1316	1418
Lb790 (pMGUAI)	897	643	537	297	191	361	264	701	584	598	407
*Lactobacillus plantarum*											
NC8	NA	NA	NA	NA	NA	NA	NA	NA	NA	NA	NA
NC8 (pSIP409UAI)	641	551	255	360	140	288	250	623	451	853	224
NC8 (pSIP411UAI)	920	960	484	137	462	653	721	1159	684	1010	162
NC8 (pMGUAI)	890	692	713	112	121	501	595	843	525	765	85
*Lactobacillus casei*											
CECT475	965	920	103	793	201	243	307	524	506	356	890
CECT475 (pSIP409UAI)	44,433	37,711	21,713	21,916	24,091	36,639	42,977	57,145	48,839	59,408	18,740
CECT475 (pSIP411UAI)	185,567	202,356	179,199	211,214	182,181	255,191	180,191	250,101	206,942	293,825	157,331
CECT475 (pMGUAI)	34,804	12,417	5619	7320	2656	4630	15,315	14,350	17,310	20,983	7379
*Enterococcus faecium*											
T136^b^	9668	4599	5555	13,419	4582	4165	3876	5663	3495	4996	5096

Most of the data are mean from two independent determinations in triplicate

*NA* no activity

^a^Antimicrobial activity expressed in BU per milligrams cell dry weight

^b^Culture of *E. faecium* T136 used as control for antimicrobial activity of EntA

### Purification of EntA and mass spectrometry analysis

**Fig. 1:**
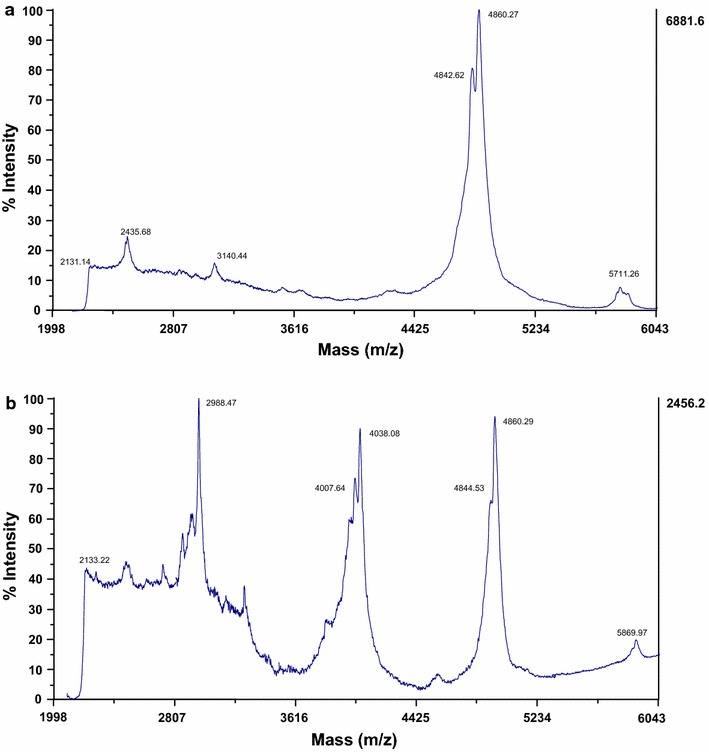
Mass spectrometry analysis of purified enterocin A from *Lb. sakei* Lb790 (pSIP411UAI) (**a**), and *Lb. casei* CECT475 (pSIP411UAI) (**b**)

The EntA produced by *Lb. sakei* Lb790 (pSIP411UAI) and *Lb. casei* CECT475 (pSIP409UAI) was purified to homogeneity following a previously described chromatographic procedure (results not shown). MALDI-TOF MS analysis of the purified EntA from *Lb. sakei* Lb790 (pSIP411UAI) showed a major peptide fragment of a molecular mass of 4842.62 Da (Fig. 1a), nearly identical to the EntA produced by different recombinant yeasts [20] while the purified EntA produced by *Lb. casei* CECT475 (pSIP411UAI) showed peptide fragments of different molecular massess among which a peptide fragment of 4844.53 Da, nearly identical to the observed molecular mass (4844.40 Da) of the EntA produced by *E. faecium* T136 [13], was also observed (Fig. 1b). In both purifications the peptide fragment of 4860.2 Da may correspond to oxidation (+16 Da) of the methionine residue (Met^33^) of the EntA to methionine sulfoxide (MetSO) (Fig. 1). The visualization by MALDI-TOF MS of peptide fragments of different molecular massess (Fig. 1b) may suggest that the EntA produced by *Lb. casei* CECT475 (pSIP411UAI) has not been purified to homogeneity or that these peptides could be responsible of the low antimicrobial activity observed in supernatants of *Lb. casei* CECT475. However, treatment of crude supernatants of the control strain *Lb. casei* CECT474 with proteinase K (1 mg/ml) revealed that the antimicrobial activity of the supernatants was not of proteinaceous nature (results not shown).

## Discussion

Lactobacilli are common colonisers of the human gastrointestinal and urogenital tracts, skin and the oral cavity and they merit recognition as starters in the production of fermented products, and as probiotics [25, 26]. They are also being evaluated for production of functional foods enriched in bioactive peptides [3]. Furthermore, production of bacteriocins by lactobacilli could find their use as natural antimicrobial peptides while the bacteriocin-producing lactobacilli could be evaluated for their improved functionality as probiotics. Several gene expression systems have been developed for efficient overproduction of heterologous proteins in LAB [1, 27, 28]. Previous studies have evaluated the production, secretion and functional expression of the EntA by different LAB, mostly of the genera *Lactococcus*, *Enterococcus*, and *Pediococcus* [13, 19, 29] and yeasts [20, 21]. However, of great biotechnological interest would be the construction of recombinant *Lactobacillus* spp. for production of bacteriocins with known and potent antimicrobial activity against *Listeria* spp.

For protein expression by *Lb. sakei and Lb. plantarum* but also for other *Lactobacillus* spp., the so-called pSIP expression vectors permits expression of the gene of interest under control of an inducible promoter by an externally added peptide pheromone [1, 28]. The pSIP system has been successfully applied for intracellular expression, secretion and surface anchoring of a variety of proteins in *Lb. plantarum and Lb. sakei* [1]. However, although these pSIP vectors have been evaluated for expression of different reporter proteins, they have not been yet fully evaluated for secretion and functional expression of bacteriocins. In these vectors the expression of genes of interest is driven by strong, regulated promoters derived from the bacteriocin sakacin P structural gene (P_*sppA*_) or the sakacin Q structural gene (P_*sppQ*_ also recorded as P_*orfX*_) with an engineered *Nco*I site for translational fusion cloning, as well as for components of the cognate two-component signal transduction system (SppK and SppR) which responds to an externally added peptide pheromone (SppIP). These vectors also carries a multicloning site (MCS) and the replicon derived from the narrow-host-range *Lactobacillus* replicon from plasmid p256 (pSIP409) or the broad-host-range, high-copy-number replicon from plasmid pSH71 (pSIP411) [28]. The expression vector pMG36c contains the low copy replication origin of plasmid pWV01 and the strong P_32_ promoter to drive the constitutive transcription of inserted genes into the multicloning site (MCS) of pUC18 [30]. Different homologous and heterologous signal peptides (SPs) have been also evaluated for secretion of heterologous proteins and bacteriocins by LAB, although expression yield and secretion efficiency are not only steered by the SP but also the host producer [1, 10, 13].

In this work, *Lb. sakei* Lb790, *Lb. plantarum* NC8 and *Lb. casei* CECT475 were transformed with the recombinant plasmids pSIP409UAI, pSIP411UAI and pMGUAI for heterologous production of EntA and evaluation of its functional expression against *Listeria* spp. The results obtained suggest that production, secretion and antimicrobial activity of the EntA produced depend on the expression vector and the host strain (Table 1). EntA producers are protected from the antagonistic effect of this bacteriocin by the concomitant expression of a cognate immunity protein (EntiA) and bacteriocins of the class IIa, such as the EntA use components of the mannose phosphotransferase system (Man-PTS) of the susceptible cells as the target/receptor. The immunity proteins form a strong complex with the receptor proteins, thereby preventing cells from being killed [15, 31]. Of interest is the 2.7- and 4.9-fold enhanced production of EntA by *Lb. sakei* Lb790 (pSIP411UAI) and *Lb. casei* CECT475 (pSIP411UAI), respectively, as compared to the rest of recombinant *Lactobacillus* spp. and *E. faecium* T136 (Table 1). The production of EntA would depend, among other factors, on plasmid stability and copy number differences between pSIP409, pSIP411 and pMG36c but, more likely, might be caused by promoters used to drive gene expression. For optimization of protein production inducible systems are often considered superior to constitutive systems since the short induction time for bacteriocin production from the pSIP-inducible vectors most probably prevents EntA from attaching to cell walls, forming aggregates, and/or undergoing protease degradation [32]. The high-copy number replicon of pSIP411 may be also a contributing factor to the higher production of EntA by *Lactobacillus* spp. recombinants transformed with pSIP411UAI instead of pSIP409UAI.

Protein secretion is a preferred means of protein expression in the development of LAB as cell factories for production of biologically active compounds [33]. However, it may happen that SP_*usp45*_ could modulate differently the secretion of EntA by the recombinant *Lb. sakei* Lb790, *Lb. plantarum* NC8 and *Lb. casei* CECT47 hosts, as it appeared with secretion of EntA and other bacteriocins by different LAB [10, 13]. It may also happen that mature EntA remain N-terminally associated to the cell membrane of the producer cells via a Sec-type signal peptide that is not cleaved off during secretion [34]. The different molecular folding of EntA inside the less EntA-producing recombinant *Lb. plantarum* hosts may also maintain the prepeptide in an secretion-incompetent conformation [35]. It is known that *Lb. plantarum* NC8 encodes three two-peptide plantaricins of narrow inhibitory spectra, regulated by a quorum sensing based network, but unable to produce bacteriocins as pure cultures in liquid media [24]. Thus, variations in bacteriocin secretion capacities may be also governed by autoinducer peptide production and recognition and post-transcriptional factors such as codon usage, mRNA stability and translational efficiency that may steer EntA production from the recombinant *Lb. sakei* Lb790 and *Lb. plantarum* NC8 [36]. New variants of the modular pSIP-vectors, encoding different SPs, have been tested for inducible gene expression and reporter protein secretion in *Lactobacillus* spp. All recombinant strains secreted the target protein nuclease A (NucA), albeit with different production levels [1].

In this work, polyclonal antibodies of predetermined specificity for EntA and an NCI-ELISA have permitted evaluation of the specific antimicrobial activity (SAA) of the produced EntA against *E. faecium* P13 (EntA^S^). From the *Lb. sakei* Lb790-derived recombinants, only *Lb. sakei* Lb790 (pSIP411UAI) showed a 2.2-fold higher antimicrobial activity (AA) but a 1.2-times lower SAA than the EntA produced by *E. faecium* T136 (Table 1). All *Lb. plantarum* NC8 recombinants showed a much lower AA and SAA when compared to the control EntA producer. However, all *Lb. casei* CECT475-derived recombinants generated supernatants with higher AA and SAA than those from *E. faecium* T136. Of interest is the 22.8-fold higher AA and the 4.7-fold higher SAA of supernatants of *Lb. casei* CECT475 (pSIP411UAI) (Table 1). According to these results, it is important to consider that not always a higher bacteriocin production by recombinant LAB may report a higher AA and SAA [9, 10]. The low AA and SAA of the EntA produced by the *Lb. sakei* Lb790- and *Lb. plantarum* NC8-hosts may depend on many factors which are difficult to determine. It is possible that: (1) regulatory responses to secretion stress activate quality control networks of the producer cells involving folding factors and housekeeping proteases [37], (2) differences in the Sec-dependent translocation and Sec-machinery, differences in protein folding, and conformational modifications of bacteriocins to a less extracellular active form may also decrease the antagonistic activity of the secreted EntA [38], (3) secretion of truncated bacteriocins may also lower the antimicrobial activity of the producer cells [10], (4) the formation of disulfide bonds (DSB) from the four cysteine residues in EntA may also play a role in the folding, structural integrity, and antimicrobial activity of the produced bacteriocin [39], and (5) the EntA contains a methionine residue that may change to an apparently less active form due to its oxidation to methionine sulfoxide [40]. The lower AA and SAA of the produced EntA may be also adscribed to differences in protein folding efficiency and bacteriocin self-aggregation [13]. Although *Lb. sakei* and *Lb. plantarum* have been considered appropriate hosts for the recombinant production of a number of reporter proteins and enzymes [1, 41–43], the results of this work resolve *Lb. casei* CECT475 as the preferred host for heterologous production and functional expression of the bacteriocin EntA.

Supernatants from all recombinant *Lb. casei* CECT475 hosts, producers of EntA, showed up to a 59.2-fold higher AA against *Listeria* spp. than any other *Lb. sakei* Lb790- or *Lb. plantarum* NC8-recombinant producer of EntA (Table 2). Furthermore, *Lb casei* CECT (pSIP411UAI) an inducible overproducer of EntA with higher AA and SAA in its supernatants than those from *E. faecium* T136, could be considered as a cellular factory and an alternative to *E. faecium* T136 for production and recovery of the highly active antilisterial bacteriocin EntA. The controlled production of EntA by *Lb. casei* CECT475 (pSIP411UAI) and the constitutive production of this bacteriocin by *Lb. casei* CECT475 (pMGUAI) could be also evaluated as a contributing antilisterial effect of *Lb. casei* CECT475, also cited as *Lb. casei* ATCC393, during further evaluation of the potential of the *Lb. casei* CECT475-derived recombinant strains during production of dry-fermented sausages [44, 45], production of antithrombotic and angiotensin converting enzyme (ACE)-inhibitory peptides (ACEIP) from bovine casein [46] or during production of antioxidant and antimutagenic peptides from yogurt [3].

## Conclusions

The use of *Lb. casei* CECT475-derived strains, generally recognized as safe (GRAS) and with a qualified presumption of safety (QPS), as recombinant bacteriocin producers may provide means by which the potential benefits of antimicrobial compounds can be exploited in the food industry, human and veterinary applications, and in the animal production field. The combined use of the inducible protein expression vector pSIP411 and *Lb. casei* CECT475 as the producer host, would also merit recognition as a novel gene expression system for the efficient overproduction and functional expression of EntA by *Lb. casei*.

## Methods

### Microbial strains, plasmids, and growth conditions

The microbial strains and plasmids used in this study are listed in Table 3. *Enterococcus faecium* T136 was used as the source of *entA* (EntA) and *entiA* (EntI), whereas *Lactococcus lactis* MG1363 was the source of the signal peptide from protein Usp45 (SP_*usp45*_). The lactococcal strains were propagated at 32 °C in M17 broth (Oxoid Ltd., Basingstoke, UK) supplemented with 0.5 % (w/v) glucose (GM17). The enterococcal strains and the lactobacilli were grown in MRS broth (Oxoid) at 32 °C. *Escherichia coli* XL10 Gold (Stratagene, La Jolla, CA, USA) was grown in BHI (Oxoid) broth at 37 °C with shaking. *Listeria* spp. strains were cultured in BHI broth (Oxoid) at 32 °C. Agar plates were made by addition of 1.5 % (w/v) agar (Oxoid) to the liquid media. When necessary, chloramphenicol (Sigma-Aldrich Inc., St. Louis, MO, USA) was added at 10 µg ml^−1^ for *E. coli*, lactococci and lactobacilli. Erythromicin (Sigma) was added at 350 µg ml^−1^ for *E. coli* and at 10 µg ml^−1^ for lactococci and lactobacilli. Cell dry weights of late exponential phase cultures expressed as cell dry mass were determined gravimetrically.

**Table 3:** Bacterial strains and plasmids used in this study

Strain or plasmid	Description^a^	Source and/or reference^b^
Strains		
*Lactobacillus sakei* Lb790	Host strain, meat isolate, non-bacteriocin producer	[22]
*Lactobacillus plantarum* NC8	Host strain, silage isolate, plasmid free	[48]
*Lactobacillus casei* CECT475	Host strain, cheese isolate, also recorded as strain ATCC393	CECT
*Lactococcus lactis* MG1363	Source of SP_*usp45*_, plasmid-free and prophage-cured derivative of *L. lactis* NCDO 712	[51]
*Enterococcus faecium* T136	Enterocin A and B producer, source of *entA* and *entiA*, control strain	DNBTA [52]
*Enterococcus faecium* P13	Enterocin P producer, control strain MPA and ADT indicator	DNBTA [52]
*Listeria ivanovii* CECT913	Indicator strain, sheep isolate	CECT
*Listeria grayi* CECT931	Indicator strain, chinchilla faeces	CECT
*Listeria welshimeri* CECT919	Indicator strain, decaying vegetation	CECT
*Listeria seeligeri* CECT917	Indicator strain, soil isolate	CECT
*Listeria innocua* CECT910	Indicator strain, cow brain isolate	CECT
*Listeria monocytogenes* CECT911	Indicator strain, spinal fluid of man	CECT
*Listeria monocytogenes* CECT935	Indicator strain, spinal fluid of child	CECT
*Listeria monocytogenes* CECT936	Indicator strain, origin not described	CECT
*Listeria monocytogenes* CECT939	Indicator strain, chicken isolate	CECT
*Listeria monocytogenes* CECT4031	Indicator strain, rabbit isolate	CECT
*Listeria monocytogenes* CECT4032	Indicator strain, soft cheese isolate	CECT
Plasmids		
pSIP409	Em^r^; pSIP401 with 256_rep_ and P_orfX_::*gusA*	[28]
pSIP411	Em^r^; pSIP401 with SH71_rep_ and P_orfX_::*gusA*	[28]
pMG36c	Cm^r^, pMG36e derivative	RUG-MG [30]
pSIP409UAI	Em^r^; pSIP409 derivative encoding the PCR product UAI (SP_*usp45*_ fused to mature *entA* and *entiA* genes)	This work
pSIP411UAI	Em^r^; pSIP411 derivative encoding the PCR product UAI (SP_*usp45*_ fused to mature *ent*A and *enti*A genes)	This work
pMGUAI	Cm^r^, pMG36c derivative encoding the SP_*usp45*_ fused to mature *entA* and *entiA* genes)	[13]

^a^ADT, agar well diffusion test; MPA, microtitre plate asay; Cm^r^, chloramphenicol resistance; Em^r^, erythromycin

^b^CECT, Colección Española de Cultivos Tipo (Valencia, Spain); DNBTA, Departamento de Nutrición, Bromatología y Tecnología de los Alimentos, Facultad de Veterinaria, Universidad Complutense de Madrid (Madrid, Spain); RUG-MG, Department of Molecular Genetics, University of Groningen (Haren, The Netherlands)

### Basic genetic techniques and enzymes

Total genomic DNA from *L. lactis* MG1363 and *E. faecium* T136 was isolated using the Wizard^®^ DNA Purification Kit (Promega, Madison, WI, USA). Plasmid DNA isolation was carried out using the QIAprep Spin Miniprep Kit (QIAGEN, Hilden, Germany), as suggested by the manufacturer, but cells were suspended with lysozyme (40 mg ml^−1^) and mutanolysin (500 U ml^−1^) and incubated at 37 °C for 10 min before following the kit instructions. DNA restriction enzymes were supplied by New England Biolabs (Beverly, MA, USA). Ligation reactions were performed with the T4 DNA ligase (Roche Molecular Biochemicals, Mannheim, Germany). *E. coli* XL10 Gold competent cells were transformed as described by the supplier (Stratagene). Competent *L. lactis* MG363 and *Lactobacillus* spp. cells were electrotransformed with a Gene Pulser^TM^ and Pulse Controller apparatus (Bio-Rad Laboratories, Hercules, CA, USA), according to Holo and Nes [47] and Aukrust and Blom [48], respectively.

### PCR amplification and nucleotide sequencing

Oligonucleotide primers were obtained from Sigma-Genosys Ltd. (Cambridge, UK). PCR-amplification of inserts was performed as previously described [13]. The PCR-generated fragments were purified by a NucleoSpin^®^ Extract II Kit (Macherey–Nagel GmbH & Co. KG, Düren, Germany) for cloning and nucleotide sequencing. Nucleotide sequencing of purified PCR products was done using the ABI PRISM^®^ BigDye^TM^ Terminator cycle sequence reaction kit and the automatic DNA sequencer ABI PRISM, model 377 (Applied Biosystems, Foster City, CA, USA), at the Unidad de Genómica (Facultad de Ciencias Biológicas, Universidad Complutense de Madrid, Madrid, Spain).

### Recombinant plasmids derived from pSIP409, pSIP411 and pMG36c

The primers and inserts used for the construction of the recombinant plasmids derived from pSIP409 and pSIP411 are listed in Table 4. Plasmid derivatives were constructed as follows: the primer pair USPNC-F/JJ8-R was used for PCR-amplification from total genomic DNA of *L. lactis* MG1363 of a 124-pb *Nco*I fragment (UA) encoding the SP_*usp45*_, with a tail complementary to the DNA encoding the N-terminal sequence of EntA. Primers JJ3-F/JJ5-R were used for PCR-amplification from total genomic DNA of *E. faecium* T136 of a 475-bp *Xho*I fragment (AI) containing mature *entA* and *entiA*. Mixtures of fragments UA and AI were used as templates to amplify the 567-bp *Nco*I/*Xho*I fragment UAI encoding the mature *entA* and *entiA* fused to the SP_*usp45*_. Fragment UAI was digested with the corresponding restriction enzymes and inserted into either pSIP409 and pSIP411, digested with *Nco*I/*Xho*I. The ligation mixtures were used to transform *E. coli* XL10 Gold and *L. lactis* MG1363 competent cells, respectively, and the selected plasmid derivatives pSIP409UAI and pSIP411UAI were checked by bacteriogenicity tests, PCR and sequencing of the inserts. The construction of plasmid pMGUAI has been described previously [13]. Plasmids pSIP409UAI, pSIP411UAI and pMGUAI were used to transform competent cells of *Lb. sakei* Lb790, *Lb. plantarum* NC8 and *Lb. casei* CECT475.

**Table 4:** Primers and PCR products used in this study

Primer or PCR product	Nucleotide sequence (5′–3′) or description	Amplification
Primers		
JJ3-F	ACCACTCATAGTGGAAAATATTATGG	AI
JJ5-R	GGCGGAGCTCTCCAGGCATTAAAATTGAGATTTATCTCCATAATC	AI, UA, UAI
USPNC-F	GAATTCTCACCATGGGAAAAAAAAAGATTATCTCAGCTATTTTAATGTCTAC	UA, UAI
JJ8-R	CCATAATATTTTCCACTATGAGTGGTAGCGTAAACACCTGACAACGG	UA
PCR products		
AI	475-bp *Xho*I fragment containing the mature enterocin A (*entA*) and immunity (*entiA*) genes	
UA	124-pb *Nco*I fragment containing the *usp45* signal peptide (SP_*usp45*_) and the begining of mature *entA*	
UAI	567-bp *Nco*I*/Xho*I fragment containing the SP_*usp45*_ fused to mature *ent*A and *entiA*	

### Antimicrobial activity of the recombinant *Lactobacillus* spp. strains

The antimicrobial activity of colonies from the recombinant *Lactobacillus* spp. strains was examined by the stab-on-agar test (SOAT), as previously described [49]. When appropriate, cultures were induced with 50 ng ml^−1^ of the inducing peptide SppIP [50] at an OD_600_ of, approximately, 0.3 and the induced cultures were grown at 30 °C for 5 h. Cell-free culture supernatants were obtained by centrifugation of cultures at 12,000×*g* at 4 °C for 10 min, adjusted to pH 6.2 with 1 M NaOH, filtered through 0.2 µm pore-size filters (Whatman Int. Ltd., Maidstone, UK), and stored at −20 °C until use. The antimicrobial activity of the supernatants was quantified by a microtiter plate assay (MPA), as previously described [13], using *E. faecium* P13 as the indicator microorganism. With the MPA, growth inhibition of the sensitive culture was measured spectrophotometrically at 620 nm with a microtitre Labsystems iEMS plate reader (Labsystems, Helsinki, Finland). One bacteriocin unit (BU) was defined as the reciprocal of the highest dilution of the bacteriocin causing 50 % growth inhibition (50 % of the turbidity of the control culture without bacteriocin). The antimicrobial activity of the recombinant *Lactobacillus* spp. hosts was also tested against selected *Listeria* spp. obtained from the CECT (Colección Española de Cultivos Tipo, Valencia, Spain), using the MPA.

### ELISA for detection and quantification of EntA

Polyclonal antibodies with predetermined specificity for EntA and a non-competitive indirect enzyme-linked inmunosorbent assay (NCI-ELISA) were used to detect and quantify EntA in supernatants of the recombinant *Lactobacillus* spp. strains, essentially as described [13]. Briefly, wells of flat-bottom polystyrene microtitre plates (Maxisorp, Nunc, Roskilde, Denmark) were coated overnight (4 °C) with supernatants from *E. faecium* T136 or the recombinant strains. After addition of the anti-EntA specific antibodies and the goat anti-rabbit immunoglobulin G peroxidase conjugate (Cappel Laboratories, West Chester, PA, USA), bound peroxidase was determined with ABTS (2,2′-azino-bis[3-ethylbenzthiazoline-6-sulfonic acid]) (Sigma) as the substrate by measuring the absorbance of the wells at 405 nm with a Labsystems iEMS reader (Labsystems) with a built-in software package for data analysis.

### Purification of EntA and mass spectrometry analyses

EntA was purified from *Lb. sakei* Lb790 (pSIP411UAI) and *Lb. casei* CECT475 (pSIP411UAI), as previously described [13]. Briefly, supernatants from early stationary phase 1-L cultures of the recombinant *Lactobacillus* spp. strains were precipitated with ammonium sulfate, desalted by gel filtration, and subjected to cation-exchange and hydrophobic-interaction chromatography, followed by reverse-phase chromatography in a fast-protein liquid chromatography system (RP-FPLC) (GE Healthcare, Barcelona, Spain). Purified fractions were subjected to matrix-assisted laser desorption/ionization time-of-flight (MALDI-TOF) mass spectrometry, as previously described [13].
